# Circular RNA 100146 functions as an oncogene through direct binding to miR-361-3p and miR-615-5p in non-small cell lung cancer

**DOI:** 10.1186/s12943-019-0943-0

**Published:** 2019-01-21

**Authors:** Lijian Chen, Aruo Nan, Nan Zhang, Yangyang Jia, Xin Li, Yihui Ling, Jiabin Dai, Shaozhu Zhang, Qiaoyuan Yang, Yanni Yi, Yiguo Jiang

**Affiliations:** 1 State Key Laboratory of Respiratory Disease, The First Affiliated Hospital of Guangzhou Medical University, Guangzhou, 510120 People’s Republic of China; 2 Institute for Chemical Carcinogenesis, Guangzhou Medical University, Guangzhou, People’s Republic of China

**Keywords:** circRNA, NSCLC, Splicing factor, microRNA

## Abstract

Circular RNAs are widely expressed in eukaryotic cells and associated with cancer. However, limited studies to date have focused on the potential role of circRNAs in progression of lung cancer. Data from the current investigation showed that circRNA 100146 is highly expressed in non-small cell lung cancer (NSCLC) cell lines and the chemically induced malignant transformed bronchial cell line, 16HBE-T, as well as 40 paired tissue samples of NSCLC. Suppression of circRNA 100146 inhibited the proliferation and invasion of cells and promoted apoptosis. Furthermore, circRNA 100146 could interact with splicing factors and bind miR-361-3p and miR-615-5p to regulate multiple downstream mRNAs. Our collective findings support a role of circRNA 100146 in the development of NSCLC and further demonstrate endogenous competition among circRNA 100146, SF3B3 and miRNAs, providing novel insights into the mechanisms underlying non-small cell lung cancer.

**Electronic supplementary material:**

The online version of this article (10.1186/s12943-019-0943-0) contains supplementary material, which is available to authorized users.

## Main text

Non-small cell lung cancer accounts for up to 85% of all lung cancer cases and is the leading cause of lung cancer-associated mortality [1]. Early diagnosis and treatment are essential to improve patient survival. However, the mechanisms underlying lung cancer progression are not yet to be fully elucidated. Recent progress in RNA research has led to the identification of non-coding RNAs (ncRNAs) involved in a variety of biological processes [2]. Previous studies have revealed critical roles of miRNAs and lncRNAs in lung cancer development, in particular, regulation of proliferation, apoptosis and invasion [3, 4]. In-depth analysis of ncRNAs should thus aid in further clarifying cancer-associated mechanisms at the epigenetic level.

Circular RNAs (circRNAs) are a class of newly discovered RNAs extensively expressed in eukaryotic cells. In recent years, the rapid development of high-throughput sequencing and bioinformatics has significantly improved our understanding of circRNAs. The majority of currently reported circRNAs are non-coding, with some have been reported to encode polypeptides or proteins [5]. CircRNAs are formed by non-canonical splicing and relatively resistant to exonuclease degradation [6]. CircRNAs are closely associated with the development of various disorders, particularly with cancer [7, 8]. However, our understanding of the specific roles of circRNAs in NSCLC remains limited and requires further exploration. In the current study, we examined the biological function of circRNA 100146 (also known as hsa_circ_0011385 in the circBase) and underlying molecular mechanisms in the development of NSCLC from multiple viewpoints. Our findings provide novel clues for the identification of biomarkers for NSCLC.

## Results and discussion

### Screening and expression of circRNA100146 in NSCLC cells and tissues

**Fig. 1:**
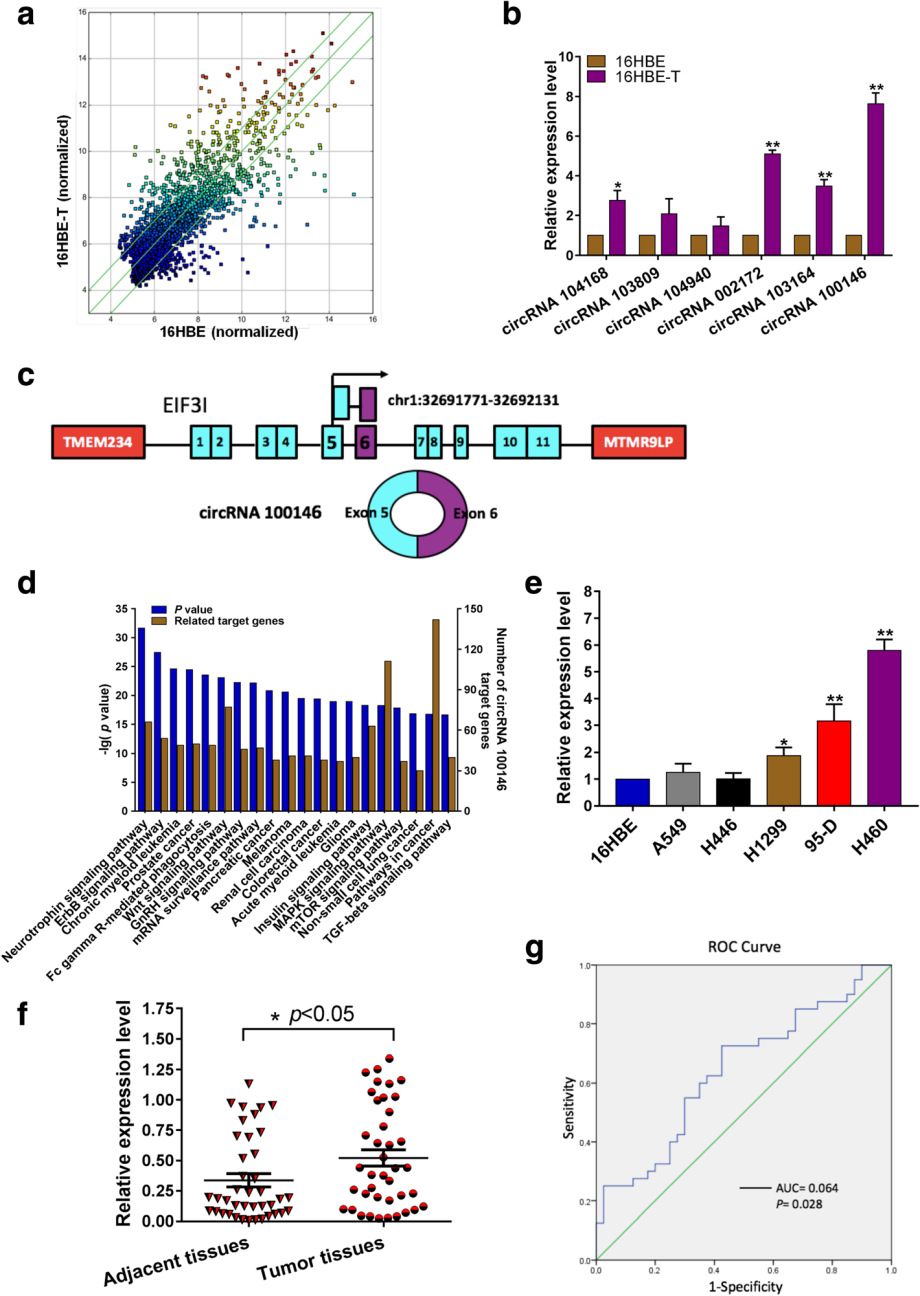
Screening and expression of circRNA100146 in NSCLC cells and tissues. **a** Differential expression circRNAs in 16HBE and 16HBE-T cells. The values plotted on X and Y axes in the Scatter-Plot are the normalized signal values of the samples (log2 scaled). The green lines are Fold Change Lines. The circRNAs above the top green line and below the bottom green line indicated circRNAs > 2.0-fold change between the two samples. **b** Six circRNAs that were mostly upregulated were detected via qRT-PCR. Data are presented as means ± s.d., *n* = 3, paired *t*-test, *p < 0.05, **p < 0.01. **c** Genomic scheme of circRNA 100146. *TMEM234* and *MTMR9LP* are genes upstream and downstream of *EIF3I*, the parental gene of circRNA 100146. **d** Pathways and cellular processes analysis were applied for prediction of circRNA 100146. We used KEGG database to analyze circRNA100146 and its target genes for signal pathway enrichment, and to calculated the hypergeometric distribution between differentially expressed genes and pathways, then analyzed the enrichment of these genes in different pathways, and finally screened out pathways that these genes involved based on the *p* value. The left Y axis represents *p* value and right Y axis represents the number of target genes associated with circRNA. **e** Relative expression of circRNA 100146 in five lung cancer cell lines. Data are expressed as means ± s.d., *n* = 3, unpaired *t*-test, *p < 0.05, **p < 0.01. **f** The scatter plot shows circRNA 100146 expression in 40 paired NSCLC cancer and paracancerous tissues (2^-ΔCt, which was normalized according to GAPDH expression level), *n* = 40, ANOVA, *p < 0.05. **g** ROC curve analysis to evaluate the diagnostic value of circRNA 100146. The area under the curve (AUC) was 0.643 (95% CI:0.521–0.764, *p = 0.028)

We employed the immortalized normal human bronchial epithelial cell line, 16HBE, and the malignantly transformed cell line, 16HBE-T, for circRNA microarray analysis (Additional file 1). Differentially expressed circRNAs were displayed through fold change filtering (Fig. 1a, Additional file 2). Specific primers were designed and qRT-PCR was employed to detect the top six upregulated circRNAs (Additional file 3: Table S1) expression in 16HBE and 16HBE-T cells. Compared with 16HBE, the expressions of circRNA 104168, circRNA 002172, circRNA 103164 and circRNA100146 were significantly higher expression in 16HBE-T cells by 2.58 ± 0.50, 5.12 ± 0.19, 3.49 ± 0.32 and 7.64 ± 0.55-fold, with circRNA 100146 displaying the greatest increase (Fig. 1b). CircRNA 100146 is formed by the head-to-tail splicing of exon 5 and exon 6 of *EIF3I*, with a predicted length of 278 bp (Fig. 1c). To validate the formation of circRNA 100146, we designed convergent primers to amplify linear mRNA, as well as divergent primers to amplify circRNA. By using cDNA and gDNA as templates, only circRNA 100146 could be amplified by divergent primers in cDNA, and no other products were observed in the gDNA groups (Additional file 4: Figure S1a). Then, the RT-PCR product of circRNA was confirmed by Sanger sequencing (Additional file 4: Figure S1b). We further predicted the involvement of circRNA 100146 in cellular processes and pathways, and found its involvement in multiple pathways, particularly pathway in cancer (Fig. 1d). Subsequently, circRNA 100146 expression was detected in lung cancer cell lines A549, H446, H1299, 95-D and H460. Compared with 16HBE, the expression of circRNA 100146 was significantly up-regulated in H1299, 95-D and H460 cells and displayed 5.80 ± 0.41-fold increase in H460 cells (Fig. 1e). We also examined circRNA 100146 expression in 40 pairs of matched NSCLC cancer and adjacent tissue samples. Compared with paracancerous tissues, circRNA 100146 was highly expressed in cancer tissue (*p* < 0.05, Fig. 1f) and upregulated in 26 cases (Additional file 4: Figure S1c). Abnormal expression of circRNA 100146 was associated with pathological classification and differentiation grade of lung cancer (Additional file 3: Table S2). We next investigated whether circRNA 100146 expression could serve as a biomarker for distinguishing cancerous tissue from adjacent non-cancerous lung tissue. ROC curve analysis was employed to evaluate the diagnostic value of circRNA 100146. Results showed that the area under the curve (AUC) was 0.643 (95% CI:0.521–0.764), and the sensitivity and specificity were 0.725 and 0.575, respectively (Fig. 1g).

### Suppression of circRNA 100146 expression inhibits cancer cell proliferation, invasion and promotes apoptosis in vitro

**Fig. 2:**
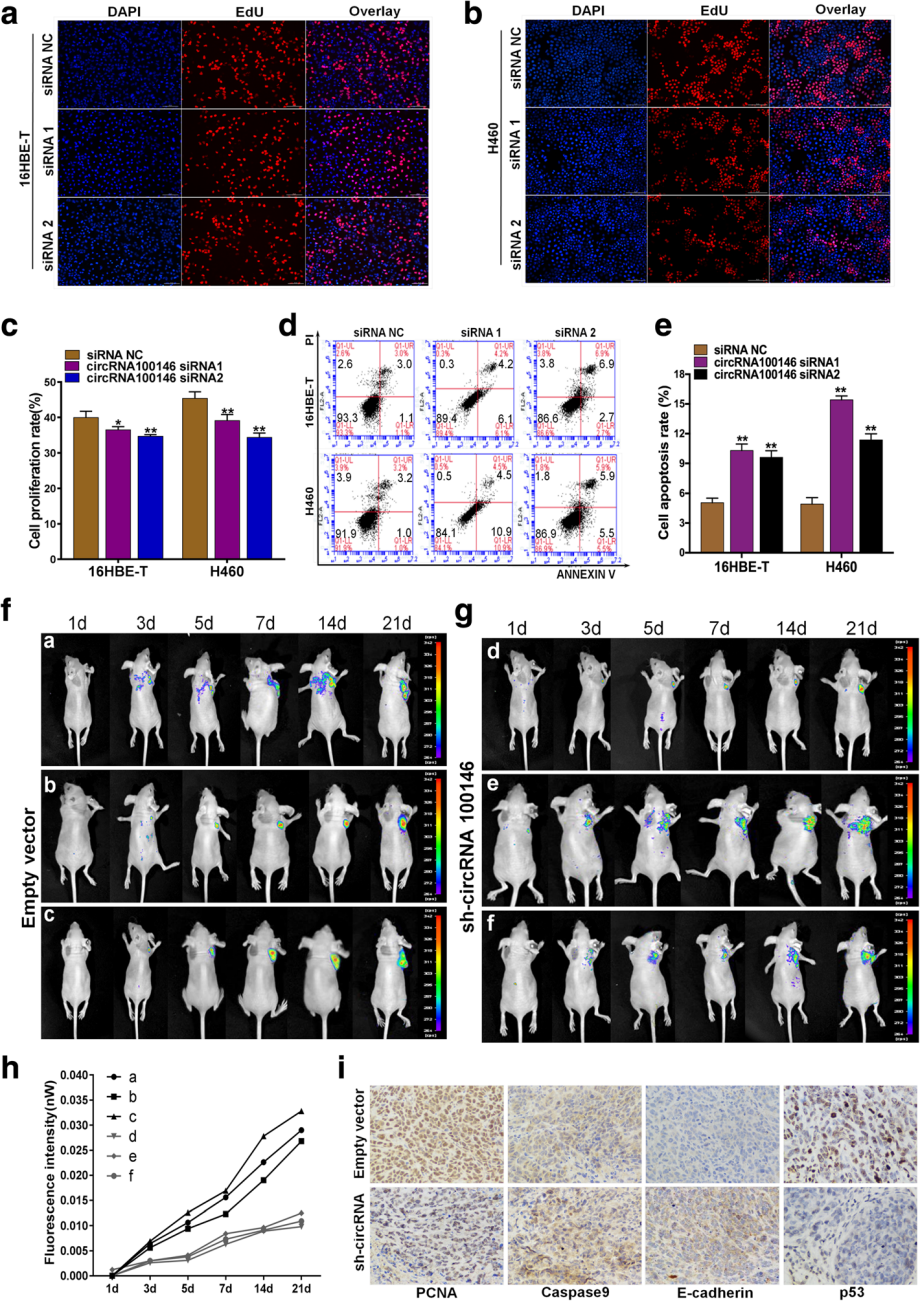
Suppression of circRNA 100146 expresssion inhibits cancer cell proliferation and promotes apoptosis in vitro and inhibits subcutaneous tumor growth in vivo*.* **a, b** After knockdown of circRNA 100146, proliferation of 16HBE-T (**a**) and H460 cells (**b**) was detected with the EdU assay. Blue fluorescence represents DAPI-stained nuclei and red fluorescence represents EdU-labeled proliferative cells (magnification× 200). **c** The histogram shows the relative proportions of EdU-positive 16HBE-T and H460 cells. Cells were counted using Image-pro-plus software. **d** Apoptosis of cancer cells was detected using flow cytometry. Upper left quadrant (UL) represents necrotic cells, lower left quadrant (LL) living cells, right upper quadrant (UR) late apoptotic and necrotic cells, lower right quadrant (LR) early apoptotic cells. **e** Percentage of apoptotic cells (sum of LR and UR) in the negative control and interference groups. **f, g** H460-empty vector(**f**) and H460-sh-circRNA(**g**) were injected subcutaneously in nude mice . Subcutaneous tumor fluorescence intensities were determined in vivo by using an small animal imaging system at 1, 3, 5, 7, 14, and 21d. There is a fluorescence value coordinate on the right side of the displayed picture, and the darker of the red, the higher of the fluorescence value. **h** Changes in subcutaneous fluorescence intensity in the empty vector and experimental groups at different time-points were analyzed. **i** Immunohistochemical detection of expression of PCNA, Caspase-9, E-cadherin and p53 in H460-empty vector and H460 sh-circRNA groups (magnification × 400). **c,e** Data are presented as means±s.d., *n* = 3, unpaired *t*-tests; compared with the siRNA NC group, *p < 0.05, **p < 0.01

To investigate specific function of circRNA 100146, we designed and synthesized three specific small interfering RNAs and examined interference efficiency. Overall, siRNA1 and siRNA2 showed the relative higher interference efficiency (*p* < 0.01; Additional file 4: Figure S2a, S2b). Notably, interference with circRNA did not affect the expression of its parental gene, *EIF3I* (Additional file 4: Figure S2c). Following 48 h transfection of 16HBE-T and H460 cells with siRNAs, cell proliferation and apoptosis were examined by 5-Ethynyl-2′-deoxyuridine (EdU) and flow cytometry (FCM) assays. Proliferation of 16HBE-T and H460 cells was significantly decreased after knockdown of circRNA 100146, compared with the siRNA NC group (*p* < 0.05, Fig. 2a-2c). FCM data revealed a marked increase in the apoptotic rates of 16HBE-T and H460 (*p* < 0.01, Fig. 2d, 2e). The effects of circRNA 100146 on invasion and migration of cancer cells were further examined via transwell, wound healing and cell adhesion assays. After transfection with siRNA1 and siRNA2, average invasion rates were (16.91 ± 0.75%) and (16.56 ± 0.85%) for 16HBE-T and (7.96 ± 0.44%) and (8.34 ± 0.79%) for H460 respectively. Compared with the control group, invasion rates were significantly reduced (*p* < 0.01; Additional file 4: Figure S2d, S2e). The wound healing assay revealed that knockdown of circRNA 100146 inhibits migration of cancer cells (*p* < 0.01; Additional file 4: Figure S2f, S2 g), analogous to data obtained with the cell adhesion assay (*p* < 0.01, Additional file 4: Figure S2 h).

### Suppression of circRNA 100,146 expression inhibits subcutaneous tumor growth in vivo

We constructed stably transfected H460 cells with knockdown of circRNA 100146. circRNA expression in H460-sh circRNA 100146 cells was depleted by 50%, compared with that in the H460-empty vector group (*p* < 0.01) (Additional file 4: Figure S2i, S2j). The relative expression of *EIF3I* was detected in H460-sh circRNA 100146 and H460-empty vector group, results showed that no statistical difference was found between the two groups (Additional file 4: Figure S2k). Xenograft nude mouse model was subsequently established using transfected H460 cells. After 21 days follow-up, growth of tumors in the sh-circRNA group was slower and tumor volumes were significantly reduced. Furthermore, we used a small animal live imaging system to obtain fluorescence images of nude mice at 1, 3, 5, 7, 14 and 21d after inoculation (Fig. 2f, 2g). Tumors in nude mice gradually grew larger and fluorescence intensity was increased with time. However, fluorescence intensity in the sh-circRNA 100146 group consistently remained weaker than that in the control group (Fig. 2h, Additional file 3: Table S3). Hematoxylin-eosin staining and immunohistochemical analysis of removed tumors disclosed decreased PCNA and p53 and increased caspase-9 and E-cadherin levels in the sh-circRNA group (Additional file 4: Figure S2i and Fig. 2i), clearly suggesting that proliferation and invasion of tumor cells are suppressed while apoptosis is enhanced.

### CircRNA 100146 binds subtypes of splicing factor SF3 family

**Fig. 3:**
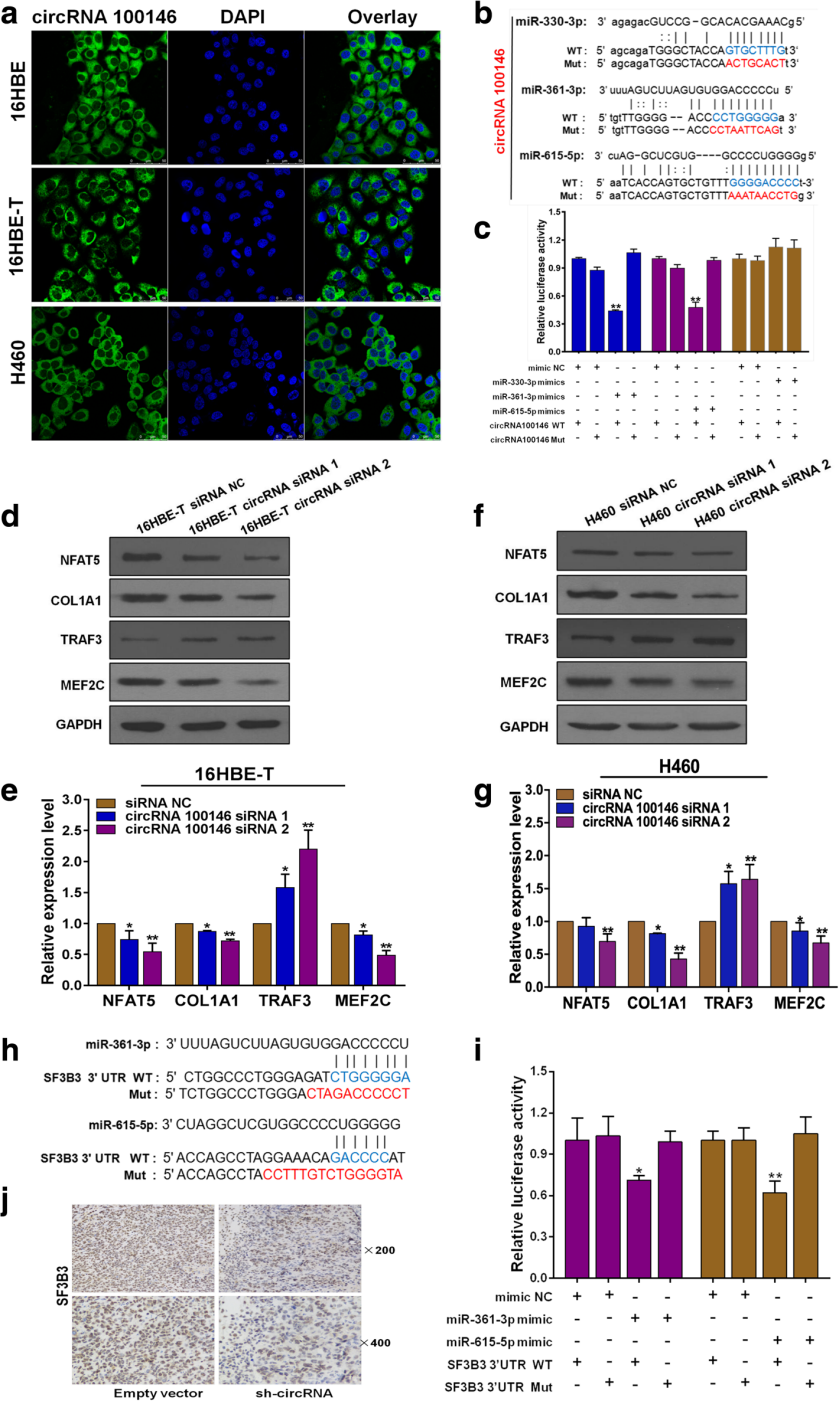
CircRNA 100146 binding to miR-361-3p and miR-615-5p indirectly affecting multiple downstream mRNAs expression. **a** Subcellular localization of circRNA 100146 detected by FISH. Images were obtained under a confocal fluorescence microscope. Green fluorescence represents 6-FAM-labeled circRNA probes while blue fluorescence represents DAPI-stained nuclei. **b** Sequence alignments between miR-361-3p, miR-615-5p or miR-330-3p, and the seed sequence of circRNA 100146. WT and Mut represent wild-type and mutant sequences of circRNA. **c** Results of the dual-luciferase reporter gene assay in HEK-293 T cells. **d, f** Western blot of NFAT5, COL1A1, TRAF3 and MEF2C in 16HBE-T (**d**) and H460 (**f**) following interference with circRNA 100146 expression. **e** Gray value analysis of the protein bands in (**d**). **g** Gray value analysis of the protein bands in (**f**). **h** Sequence alignments between miRNAs and seed sequence of the 3’-UTR of *SF3B3*. **i** Results of the dual-luciferase reporter gene assay. **j** Expression of SF3B3 in subcutaneous tumors tissues detected using immunohistochemistry. **c, i** Data are presented as means ± s.d, n = 3, unpaired *t*-test, compared with the mimic NC + WT group, *p < 0.05, **p < 0.01. **e, g** Data are presented as means ± s.d, n = 3, unpaired *t*-test, Compared with siRNA NC group, *p < 0.05, **p < 0.01

To clarify circRNA 100146 expression at the subcellular level, fluorescence in situ hybridization (FISH) was performed in 16HBE, 16HBE-T and H460 cells. The overlay images showed expression of circRNA was mainly in the cytoplasm (Fig. 3a). Moreover, nuclear and cytoplasmic RNA was extracted from cells and circRNA relative expression was detected by qRT-PCR and results also showed circRNA 100146 was predominantly expressed in the cytoplasm (Additional file 4: Figure S3a). To determine the possible mechanisms underlying the effects of circRNA 100146 on general transcription, we designed specific circRNA 100146 protein-pull-down probe and isolated the interacting proteins in H460 cells (Additional file 4: Figure S3b). Protein mass spectrometry facilitated the identification of 657 proteins in total. Gene ontology analysis further disclosed that nucleic acid binding proteins accounted for 31.2% of all the identified proteins (Additional file 4: Figure S3c and Additional file 5). Upon ranking of the proteins according to score (high to low), we observed that the splicing factor SF3 family was the most closely associated with gene transcription among the top 100 proteins, including SF3B3, SF3B2 and SF3A1 (Additional file 4: Figure S3d). Our findings indicate that circRNA 100146 bind to multiple subtypes of splicing factor family SF3.

### CircRNA 100146 direct binding to miR-361-3p and miR-615-5p

Several studies have reported circRNAs can act as miRNA “sponge” affecting miRNA activity [9]. We further explored regulatory functions of circRNA 100146 at post-transcriptional level. Except for bioinformatics analysis, we also performed an RNA antisense purification experiment (Additional file 4: Figure S4a) and sequenced the products to accurately identify interacting miRNAs. Overall, miRNAs accounted for 7.26% of the total non-coding RNAs (Additional file 4: Figure S4b). Based on bioinformatics, sequencing and literature reviews, miR-330-3p, miR-361-3p and miR-615-5p that directly bind to circRNA 100146 were selected for further analyses (Additional file 4: Figure S4c). Furthermore, dual luciferase reporter assays were used to detect direct interactions between circRNA 100146 and three miRNAs. Co-transfection cells with circRNA wild-type vector and miR-361-3p or miR-615-5p mimics led to significantly reduced relative luciferase activity while co-transfection with miR-330-3p mimics induced non-significant differences (Fig. 3b, 3c), clearly suggesting that circRNA 100146 interacts directly with miR-361-3p and miR-615-5p. Moreover, FISH experiments showed that circRNA 100146, miR-361-3p and miR-615-5p are co-expressed in the cytoplasm at the subcellular level (Additional file 4: Figure S4d). Our results support that circRNA 100146 direct binding to miR-361-3p and miR-615-5p to regulating their activity. In view of the association of two miRNAs with NSCLC development, we propose that circRNA 100146 affects tumor progression via regulation of miR-361-3p and miR-615-5p.

### CircRNA 100146 indirectly affects multiple downstream mRNAs through regulating miR-361-3p and miR-615-5p

We used miRDB and TargetScan to predict the target mRNAs of miR-361-3p or miR-615-5p. Combination of sequence matching, functional analyses and correlations with cancer led to the identification of 21 mRNAs. Twelve of these were targeted by miR-361-3p and 9 by miR-615-5p (Additional file 3: Table S4). Upon knockdown of circRNA 100146 or overexpression of miR-361-3p or miR-615-5p in 16HBE-T and H460, some mRNAs were significantly downregulated, including *NFAT5*, *COL1A1*, *TRAF3* and *MEF2C* (Additional file 4: Figure S4e-S4 h). Bioinformatics analyses suggested that *TRAF3*, *NFAT5* and *COL1A1* are likely to be the direct targets of miR-361-3p and *MEF2C* the direct target of miR-615-5p. Following suppression of circRNA 100146, we detected endogenous proteins expression of NFAT5, COL1A1, MEF2C and TRAF3 via western blot. Result showed NFAT5, COL1A1 and MEF2C proteins were downregulated and TRAF3 protein upregulated in16HBE-T cells (Fig. 3d, 3e). Endogenous proteins were expressed to a similar extent in H460 cells (Fig. 3f, 3g). In summary, changes of circRNA 100146 expression affected multiple downstream genes through regulating miR-361-3p and miR-615-5p.

Splicing factor, *SF3B3*, is predicted to be a common target of miR-361-3p and miR-615-5p (Additional file 4: Figure S4i). Upon overexpression of miR-361-3p or miR-615-5p, SF3B3 expression was decreased (*p* < 0.01, Additional file 4: Figure S4j). We further conducted dual luciferase reporter gene assay with plasmids expressing wild-type and mutant SF3B3 3’UTR (Fig. 3h). Data showed that miR-361-3p directly binds to the 3’UTR region of *SF3B3* whereas only one of the two predicted sites of miR-615-5p binds directly to this region (Fig. 3i). Western blot showed SF3B3 protein are reduced upon overexpression of miR-361-3p or miR-615-5p in H460 cells (Additional file 4: Figure S4k, S4 l). The relative expression of *SF3B3* was detected in H460-sh circRNA and H460-empty vector, results show that that *SF3B3* expression was decreased in H460-sh circRNA group (*p* < 0.01, Additional file 4: Figure S4 m). Immuno-histochemical analysis of SF3B3 expression in subcutaneously transplanted tumors revealed significantly decreased in the sh-circRNA group (Fig. 3j). Based on this, we propose that circRNA 100146 may affect *SF3B3* expression through regulating miR-361-3p and miR-615-5p.

## Conclusions

We have demonstrated an oncogenic role of circRNA 100146 in the progession of NSCLC for the first time. The endogenous competitive relationships among circRNA 100146, SF3B3 and miRNAs has been elaborated. Our data provide a platform for further elucidating mechanisms underlying the NSCLC and identifying diagnostic or therapeutic targets.

## Additional files

Additional file 1: Supplementary materials and methods. (DOCX 149 kb)

Additional file 2: Microarray analysis of up-regulated circRNAs. (XLS 765 kb)

Additional file 3: Supplementary tables (**Table S1-S11.**) (DOCX 45 kb)

Additional file 4: **Figure S1.** Identification of circRNA 100146 and its expression in lung cancer tissues. **Figure S2.** Suppression of circRNA 100146 expresssion inhibits cancer cell invasion and migration in vitro. **Figure S3.** circRNA 100146 binds subtypes of splicing factor SF3 family. **Figure S4.** circRNA 100146 binding to miR-361-3p and miR-615-5p indirectly affecting multiple downstream mRNAs expression. (DOCX 4346 kb)

Additional file 5: Excel document with protein mass spectrum of pull down experiment. (XLS 6165 kb)

## Abbreviations

Circular RNA: CircRNA
COL1A1: Collagen type I alpha 1 chain
EdU: 5-Ethynyl-2′-deoxyuridine
FCM: Flow Cytometry
FISH: Fuorescence in situ hybridization
HBE: Human Bronchial Epithelial
MEF2C: Myocyte enhancer factor 2C
NFAT5: Nuclear factor of activated T-cells 5
NSCLC: Non-small cell lung cancer
RAP: RNA antisense purification
SF3B3: Splicing factor 3b subunit 3
TRAF3: TNF receptor associated factor 3
