# Supplementary material for: Circular RNA 100146 functions as an oncogene through direct binding to miR-361-3p and miR-615-5p in non-small cell lung cancer
Source: Mol Cancer. 2019 Jan 21;18:13. doi: 10.1186/s12943-019-0943-0 (PMC6340182; doi:10.1186/s12943-019-0943-0)
Supplement: Supplementary file 1 — Supplementary materials and methods. (DOCX 149 kb) [file 12943_2019_943_MOESM1_ESM.docx]

**Materials and Methods**

**circRNA Array analysis**

The Arraystar Human circRNA Array analysis of this study was detected by Kangcheng Bio. (Shanghai, China). Total RNA from each sample was quantified using the NanoDrop ND-1000. The sample preparation and microarray hybridization were performed based on the Arraystar’s standard protocols. Briefly, total RNAs were digested with Rnase R (Epicentre, Madison, WI, USA) to remove linear RNAs and enrich circular RNAs. Then, the enriched circular RNAs were amplified and transcribed into fluorescent cRNA utilizing a random priming method (Arraystar Super RNA Labeling Kit; Arraystar). The labeled cRNAs were hybridized onto the Arraystar Human circRNA Array (8x15K, Arraystar). After washing the slides, the arrays were scanned by the Agilent Scanner G2505C. Agilent Feature Extraction software (version 11.0.1.1) was used to analyze acquired array images. Quantile normalization and subsequent data processing were performed using a R software package. Differentially expressed circRNAs between 16HBE and 16HBE-T samples were identified through Fold Change filtering.

**Cell lines and tissue samples**

16HBE is an immortalized normal human bronchial epithelial cell line while 16HBE-T is a malignantly transformed lung cancer cell line induced by benzo[a]pyrene-trans-7,8-diol-9,10 epoxide, established earlier by our laboratory [1]. Lung cancer cell lines, A549, H446, H1299, 95-D and H460, obtained from the cell bank of the Institute for Chemical Carcinogenesis, Guangzhou Medical University, were subjected to STR genotyping and compared with data from the American Type Culture Collection. HEK-293T cells were used for dual-luciferase reporter gene assay. 40 NSCLC and matched paracancerous tissue samples were collected from the General Hospital of Guangzhou Military Command of PLA. All tissue samples were stored in liquid nitrogen. Informed consent was obtained from all patients before sample collection. This study was approved by the Ethnic Committee of the General Hospital of Guangzhou Military Command of PLA.

**RNA extraction and qRT-PCR**

Total RNA was extracted with TRIzol reagent (Invitrogen, Carlsbad, CA, USA) and quantitated using a NanoDrop1000 Spectrophotometer (NanoDrop, Wilmington, USA). Samples were incubated with RNase R (3U/µg) at 37^0^C for 15 min to degrade linear RNA before reverse transcription. Reverse transcription and PCR of circRNAs and mRNAs were conducted using the Goscript^TM^ Reverse Transcription System and GoTaq^®^ qPCR Master Mix (Promega, Madison, WI, USA) with GAPDH as an internal control. When reverse transcription of circRNA, only random primer was added, oligo-d(T) was not. An All-in-One^TM^ miRNA qRT-PCR Detection Kit (Genecopoeia, Rockville, MD, USA) was employed for miRNAs with U6 as the internal reference. Experiments were repeated three or more times. Relative expression of genes in cells was calculated using the 2^-△△Ct^ method, and relative expression of circRNA100146 in lung cancer tissues was calculated using the 2^-△Ct^ method. All primer sequences used in this study are presented in Additional file 3: Table S5.

**Sanger sequencing**

The amplification products were inserted into a T vector for Sanger sequencing to determine their full-length. The divergent primers were designed to confirm the back-splice junction of circRNA 100146: 5’-GAGCTCAACCAGTATAGTGCC-3’ (sense) and 5’-ACATGATGATGTTGCCCCCAA-3’(antisense). The primers were synthesized by Invitrogen (Shanghai, China), and Sanger sequencing was performed by Sangon Biotech (Guangzhou, China).

**RNA interference and overexpression**

We designed three siRNA pairs (RIBOBIO, Guangzhou, China) based on the circRNA100146 sequence (in Additional file 3:Table S6), which were used to transfect 16HBE-T and H460 cells for transient interference with circRNA100146 expression. Conversely, microRNA mimics (RIBOBIO) were used for overexpression of miRNAs. Lipofectamine^TM^2000 (Invitrogen) and the Ribo FECT^TM^ CP Transfection Kit (RIBOBIO) were used for transfection. All experiments were performed in triplicate.

**Cell proliferation and apoptosis**

The Cell-Light^TM^ EdU Kit (RIBOBIO) was used to determine the relative cell growth and viability of each group according to the manufacturer's protocol. Images of cells were obtained under a fluorescence microscope (Leica, Germany). The FITC-Annexin V Apoptosis Detection Kit (KeyGen Biotech, Nanjing, China) was used to evaluate apoptosis. Cells (1-5 ×10^5^) were resuspended in 100 μL 1×Binding Buffer and incubated with Annexin V (5 µL) and PI staining solution (5 µL) in the dark at room temperature for 10 min. Cells were analyzed using flow cytometry (BD Biosciences, USA) within 1 h. All experiments were performed in triplicate.

**Cell migration, invasion and adhesion**

The wound healing assay was performed to assess cell migration. Cells were seeded into 6-well plates and transfected after overnight incubation. After 6 h, vertical lines were scratched across the center of the well. Cells were incubated with serum-free medium for 48 h. Image J software (National Institutes of Health, Bethesda, MD) was used for statistical analysis of migration. Cell invasion was assessed using the transwell assay (Corning Incorporated, Corning, NY, USA) according to previous protocols [2]. The CCK-8 Kit (Beyotime, Shanghai,China) was used to detect cell adhesion. A 96-well plate was preloaded with Matrigel (10 µg/mL) and incubated at 4^0^C overnight, followed by blocking with 1% BSA. Transfected cells were seeded into the plate and cultured at 37^0^C for 1 h. Each well was incubated with CCK-8 (10 µL) for 2 h and absorbance measurements conducted at 450 nm using a microplate reader (BioTex, Houston, Texas). Experiments were performed in triplicate.

**Plasmid construction and stable transfection**

We achieved stable knockdown of circRNA 100146 expression with the aid of a specific short hairpin RNA (shRNA) sequence ( in Additional file 3:Table S7). Double-stranded DNA was dissolved in annealing buffer. The DNA solution was added in a 1.5 mL centrifuge tube, mixed evenly and incubated at 95^0^C for 2 min and 25^0^C for 45 min, followed by slow cooling to 4^0^C. DNA samples were double-digested with *Bam*HI and *Eco*RI. Annealed DNA fragments were ligated into plVX vectors and subjected to competent cell transformation and extraction of plasmids without endotoxin. The recombinant plasmid was verified via DNA sequencing (Hiseq 2000, Illumina, USA). H460 cells were transfected with extracted plasmids from each group and designated 'H460-empty vector' and 'H460-sh circRNA 100146', respectively.

**Xenograft nude mouse model**

Female BALB/c nude mice (4 weeks old) were purchased from the Medical Animal Experimental Center of Guangdong Province (Guangzhou, China) and randomly assigned to experimental and control groups. Each group contained three mice. H460 cells stably transfected with sh-circRNA 100146 or empty vector were resuspended in PBS. The cell suspension (100 μL; 1×10^8^ cells/ml) was injected into the right armpit of nude mice and the small animal live imaging system (Berthold Technologies, UK) employed to record subcutaneous tumor growth. Fluorescent images were obtained at 1, 3, 5, 7,14 and 21 d. Nude mice were sacrificed after 21 d, following which tumors were removed and subjected to pathologic and immunofluorescence analyses. Experiments were approved by the Institutional Animal Care and Use Committee of Guangzhou Medical University.

**Fluorescence *in situ* hybridization (FISH)**

FISH was performed to detect the presence of circRNA 100146, miR-361-3p and miR-615-5p. The specific probe sequences used (Sangon Biotech, Shanghai, China) are presented in Additional file 3:Table S8. Cells seeded on coverslips were fixed with 4% paraformaldehyde at room temperature for 20 min, and digested with protease K (Sangon Biotech) at 37^0^C for 5 min. Next, cells were fixed with 1% paraformaldehyde for 10 min and dehydrated over a gradient of 70%, 85% and 100% alcohol. The probe hybrid solution was dropped on the coverslip, mounted and denatured at 73^0^C for 3 min. Hybridization lasted for 12-16 h at 37^0^C in the dark. Coverslips were countstained with DAPI and mounted with an anti-fluorescent decay reagent. Cells were examined under a confocal microscope (Leica, Mannheim, Germany) and images obtained.

**RNA pull-down assay**

The BersinBio^TM^ RNA pulldown kit (BersinBio, Guangzhou*,* China) was used to detect interactions between circRNA 100146 and proteins. The biotin-labeled probe sequence is shown in Additional file 3:Table S9. The probe solution (4 μg) was denatured at 90°C for 2 min and incubated with pre-cooled RNA structure buffer to form RNA secondary structures. Next, streptavidin magnetic beads (Invitrogen) were incubated with the mixture at 25^0^C for 30 min. Cells were lysed at 4℃ and centrifuged with 15000g for 15min, supernatant fractions collected and mixed with the probe-bead mixture. RNase inhibitor (5 μL), EDTA (5 μL) and EGTA (2.5 μL) were added, followed by incubation for 2 h and elution at 37^0^C for 2 h. The supernatant fractions were transferred into a new tube and used for protein mass spectrometry.

**RNA antisense purification**

The RNA antisense purification kit (BersinBio) was used to detect RNAs-circRNA interactions via specific biotin-labeled circRNA probes. 1×10^7^ cells were washed with PBS, UV-crosslinked for 1 min and lysed with 1 mL lysis buffer. A biotin-labeled 50 bp antisense probe (in Additional file 3:Table S10) targeting the back-splice junction of circRNA 100146 was added, followed by denaturation at 65^0^C for 10 min and hybridization at room temperature for 2 h. Streptavidin magnetic beads were added (200 μL), non-specific binding RNAs removed by washing with elution buffer. Then enriched RNAs interacting with circRNA were collected and transcribed to cDNAs and identified via sequencing (ABI 3730, Applied Biosystems, Shanghai). The microRNA data obtained from sequencing have been presented in Additional file 3: Table S11.

**Dual-luciferase reporter assay**

The dual-luciferase reporter system (Promega) was employed for gene detection according to the manufacturer's instructions. CircRNA 100146 and SF3B3 3’UTR wild-type and mutant vectors were constructed using psiCheck and pmirGLO, respectively. HEK-293T cells were co-transfected with wild-type or mutant vectors and mimics using Lipofectamine^TM^2000 reagent. PLB cell lysis solution was added to each well after 48 h culture. Cells were fully lysed and the supernatant transferred into new tubes. PLB sample (20 μL) and LAR (100 μL) were added to each well of the 96-well plate, and Relative Light Units measured. After the addition of 100 μL Stop&Glo^®^ Reagent into the same well, RLU values were re-analyzed.

**Western blot**

Following extraction of total proteins, 50 μg/well protein was separated on 12% SDS gels and transferred to polyvinylidene fluoride membrane (Millipore, Billerica, MA, USA) using a standard wet transfer device (Bio-Rad). Blots were incubated with the primary antibodies, anti-SF3B3, NFAT5, COL1A1,TRAF3, and MEF2C (Abcam, Shanghai, China), followed by secondary antibodies, HRP goat anti-mouse and HRP goat anti-rabbit antibody (BOSTER Biological Technology, Wuhan, China) using GAPDH as the control. The dilution and incubation time of the antibodies are operated following the recommended instructions. BeyoECL-Plus chemiluminescent reagent (Beyotime) was used for staining and imaging.

**Bioinformatics Analysis**

To get insight into the biological functions affected by circRNA 100146, KEGG (Kyoto Encyclopedia of Gene and Genomes) Pathway Database was used for enrichment analysis of circRNA 100146 (http://www.genome.jp/kegg/pathway.html). The hypergeometric test was applied for calculate p values of target genes against the background genes in the associated pathway. RegRNA (http://regrna.mbc.nctu.edu.tw/html/prediction.html) was used for prediction of the circRNA-targeted microRNA by circRNA sequence. Targetscan（http://www.targetscan.org/vert_72/）was applied for prediction of interaction between mRNAs and microRNAs.

**Statistical analysis**

SPSS 19.0 (IBM, Armonk, NY, USA) was employed for statistical analysis, and images were plotted using GraphPad Prism 7.0 (GraphPad Software, CA, USA). The paired or unpaired *t*-test was used for comparison between two groups and one-way ANOVA for more than two groups. *P*<0.05 were considered as statistically difference.

**References**

1. Jiang Y, Wu Y, Greenlee AR, Wu J, Han Z, Li X, Zhao Y. miR-106a-mediated malignant transformation of cells induced by anti-benzo[a]pyrene-trans-7,8-diol-9,10-epoxide. Toxicol Sci. 2011;119(1):50-60.
2. Yang Q, Zhang S, Liu H, Wu J, Xu E, Peng B, Jiang Y. Oncogenic role of long noncoding RNA AF118081 in anti-benzo[a]pyrene-trans-7,8-dihydrodiol-9,10-epoxide-transformed 16HBE cells. Toxicol Lett. 2014;229(3):430-9.
