# Supplementary material for: Circular RNA 100146 functions as an oncogene through direct binding to miR-361-3p and miR-615-5p in non-small cell lung cancer
Source: Mol Cancer. 2019 Jan 21;18:13. doi: 10.1186/s12943-019-0943-0 (PMC6340182; doi:10.1186/s12943-019-0943-0)
Supplement: Supplementary file 3 — Supplementary tables (Table S1-S11.) (DOCX 45 kb) [file 12943_2019_943_MOESM3_ESM.docx]

**Supplementary Tables**

**Table S1 The top six upregulated circRNAs**

| **CircRNA** | **type** | **Gene symbol** | **Chrom** | **Spliced sequence length** | **Cyclized exon** |
| --- | --- | --- | --- | --- | --- |
| hsa_circRNA_100146 | exonic | EIF3I | chr1(+):32691771-32692131 | 278 | 5-6 |
| hsa_circRNA_104168 | exonic | RTN4IP1 | chr6(-):107031202-107050797 | 463 | 6-9 |
| hsa_circRNA_103809 | exonic | ZFR | chr5(-):32379220-32388780 | 693 | 13-17 |
| hsa_circRNA_104940 | exonic | NUP214 | chr9(+):134011326-134022971 | 1102 | 9-14 |
| hsa_circRNA_103164 | exonic | PI4KA | chr22(-):21158587-21159453 | 293 | 11-12 |
| hsa_circRNA_002172 | intragenic | RPPH1 | chr14(-):20811305-20811436 | 131 |  |

| **Table S2 Correlations between circRNA 100146 and clinicopathological parameters of lung cancer tissues** | | | | |
| --- | --- | --- | --- | --- |
| **Clinicopathological parameters** | **No. of cases** | **Relative expression** | | ***P* value** |
|  |  | **High** | **Low** |  |
| Gender |  |  |  |  |
| male | 22 | 15（57.7%） | 7（50.0%） | 0.641 |
| female | 18 | 11（42.3%） | 7（50.0%） |  |
| Age |  |  |  |  |
| ≥60 | 24 | 13（50.0%） | 11（78.6%） | 0.079 |
| ＜60 | 16 | 13（50.0%） | 3 （21.4%） |  |
| Differentiation |  |  |  |  |
| well | 9 | 8 （30.8%） | 1（7.10%） | 0.029* |
| moderate | 16 | 12（46.2%） | 4（28.6%） |  |
| poor | 15 | 6 （23.1%） | 9（64.3%） |  |
| Lymphatic metastasis |  |  |  |  |
| positive | 20 | 12（46.2%） | 8（57.1%） | 0.507 |
| negative | 20 | 14（53.8%） | 6（42.9%） |  |
| Primary location |  |  |  |  |
| left lung | 16 | 9 （34.6%） | 7（50.0%） | 0.343 |
| right lung | 24 | 17（65.4%） | 7（50.0%） |  |
| History type |  |  |  |  |
| adenocarcinoma | 23 | 13（50.0%） | 10（71.4%） | 0.001* |
| squamous | 12 | 11（42.3%） | 1 （7.10%） |  |
| others | 5 | 2 （7.70%） | 3 （21.4%） |  |
| TNM stage |  |  |  |  |
| Ⅰ | 11 | 7 （28.0%） | 4 （26.7%） | 0.927 |
| Ⅱ/Ⅲ/Ⅳ | 29 | 18（72.0%） | 11（73.3%） |  |
| Smoking history |  |  |  |  |
| smokers | 17 | 13（48.1%） | 4（30.8%） | 0.298 |
| never smokers | 23 | 14（51.9%） | 9（69.2%） |  |
| * *P*<0.05 |  |  |  |  |

**Table S3**

| **Table S3-1 Average Fluorescence Intensity of Empty vector group (nW)** | | | |
| --- | --- | --- | --- |
| Date | a | b | c |
| 1d | 0.0000 | 0.0000 | 0.0000 |
| 3d | 0.0064 | 0.0056 | 0.0069 |
| 5d | 0.0106 | 0.0094 | 0.0126 |
| 7d | 0.0156 | 0.0123 | 0.0169 |
| 14d | 0.0226 | 0.0190 | 0.0278 |
| 21d | 0.0290 | 0.0268 | 0.0328 |
| **Table S3-2 Average Fluorescence Intensity of sh-circRNA 100146 group (nW)** | | | |
| Date | d | e | f |
| 1d | 0.0000 | 0.0012 | 0.0000 |
| 3d | 0.0026 | 0.0030 | 0.0030 |
| 5d | 0.0031 | 0.0041 | 0.0038 |
| 7d | 0.0063 | 0.0084 | 0.0073 |
| 14d | 0.0089 | 0.0096 | 0.0091 |
| 21d | 0.0098 | 0.0125 | 0.0109 |

| **Table S4**  **Table S4-1 Target genes of miR-361-3p** | |
| --- | --- |
| ARNT | aryl hydrocarbon receptor nuclear translocator |
| EP300 | E1A binding protein p300 |
| RARA | retinoic acid receptor, alpha |
| TRAF3 | TNF receptor-associated factor 3 |
| BTRC | beta-transducin repeat containing E3 ubiquitin protein ligase |
| NFAT5 | nuclear factor of activated T-cells 5, tonicity-responsive |
| PPP3CA | protein phosphatase 3, catalytic subunit, alpha isozyme |
| ELK1 | ELK1, member of the ETS oncogene family |
| RASGRP2 | RAS guanyl releasing protein 2 (calcium and DAG-regulated) |
| TAOK2 | TAO kinase 2 |
| COL1A1 | collagen, type I, alpha 1 |
| SRC | SRC proto-oncogene, non-receptor tyrosine kinase |

| **Table S4-2 Target genes of miR-615-5p** | |
| --- | --- |
| COL6A2 | collagen, type VI, alpha 2 |
| MAPK10 | mitogen-activated protein kinase 10 |
| MAPK9 | mitogen-activated protein kinase 9 |
| MEF2C | myocyte enhancer factor 2C |
| NR4A1 | nuclear receptor subfamily 4, group A, member 1 |
| PPP5C | protein phosphatase 5, catalytic subunit |
| APH1A | APH1A gamma secretase subunit |
| TPM3 | tropomyosin 3 |
| PPP2R5C | protein phosphatase 2, regulatory subunit B', gamma |

**Table S5 Primer sequences for quantitative real-time PCR (5′to 3′)**

| **Gene** | **Forward primer** | **Reverse primer** |
| --- | --- | --- |
| hsa_circRNA 104168 | GAGTCCATTATCGCTGGGCA | GCAAAAGTACCAACTCCGCC |
| hsa_circRNA 103809 | TTCGAGACCTCTGTCAGCGA | ATCAGATGAGTCAGGACGACG |
| hsa_circRNA 104940 | TCTGCTCAGGGCAGTTCAAG | ACTTCTGTTGAAGCCGCAGA |
| hsa_circRNA 002172 | AGCTTGGAACAGACTCACGG | TCCTGCCCAGTCTGACCTC |
| hsa_circRNA 103164 | ATCTGAGAAGCTGCAGTCCA | TGCTCCAGCACAAAATCATGG |
| hsa_circRNA 100146 | GAGCTCAACCAGTATAGTGCC | ACATGATGATGTTGCCCCCAA |
| GAPDH | AGAAGGCTGGGCTCATTTG | AGGGGCCATCCACAGTCTTC |
| NFAT5 | GCAGGTTTCCTTGTTGCAGA | GTCTGCTTCCTCCCAGTTCT |
| COL1A1 | AGGCATCACAACAACAGCAG | GATAACTGCTGCTGTGCCTC |
| TRAF3 | GCTACTACCGGGCTGATGAT | ACCAGTCTCCATGTTGCAGA |
| MEF2C | GGTATGGCAATCCCCGAAAC | TGGCATCGTATTCTTGCTGC |
| EIF3I | CTGTGGCCAAGGACCCTATC | CCAGTGAGGACATGCTTGGT |
| SF3B3 | CTGGTGATGTCTGGGCATGT | TAACAGGGCCCCTCAGCTAT |
| PITX2-3 | GCAGATCTGGTCCGTCGCT | CGATTTCTTCGCGTGTGGAC |
| PITX2-4 | TTATTAGGGCTGCCGGGTTG | TGCCGATTCGATCCTTGTGG |
| PITX2-5 | GCCACTGAGAGATAACGGGG | GAGAGTCCGTGAACTCGACC |
| PITX2-6 | CTCTCTCCCGGTAGCCGATA | AGAGTCCGTGAACTCGACCT |
| MICAL2-2 | TGCCTGAGAATCCAGTGTGAC | CCAGAGGTGTAGCACGTTGT |
| MICAL2-3 | AACCCGTGTGTGTCTCATCC | CCAGAGGTGTAGCACGTTGT |
| MICAL2-5 | GAAGTCACCTTCAGGGTTCCA | ACCCAGTTGCAGATGCTCTC |
| MICAL2-12 | TGCCTGAGAATCCAGTGTGAC | CCAGAGGTGTAGCACGTTGT |
| MICAL2-15 | CTGCAGACTTTGCCACCAAC | GGTCCAACGTGTACTGCTCA |
| CDKN1C-1 | CACCGGACAGCCAGGTAGC | GCTTGGCGAAGAAATCGGAGA |
| CDKN1C-2 | GCGGTGAGCCAATTTAGAGC | AGATGGAGAGTGCCTTTGGC |
| PPP2R1B-1 | TGCCTTATATAAAGTGTCCTGAC | GGTGCCTGATTCCACAGTGA |
| PPP2R1B-2 | TGTCTGCTGGATTCAGTGCG | CAGCTTCTCGGATGGCGTAT |
| PPP2R1B-4 | ACTCCTGTTGCTCTGGAAGC | GCGAACGCGCCAAGATTTAT |
| U6 | CTCGCTTCGGCAGCACA | AACGCTTCACGAATTTGCGT |
| hsa_miR-361-3p | TCCCCCAGGTGTGATTCTGATTT |  |
| hsa_miR-615-5p | GGGGGTCCCCGGTGCTCGGAC |  |

**Table S6 Sequences of siRNAs for knockdown of hsa_circRNA_100146 expression**

| **Target Gene** | **Type** |  | **siRNA Sequence** |
| --- | --- | --- | --- |
| circRNA 100146 | siRNA 1 | sense | GUAUAGUGCCAAGGAAAGC dTdT |
|  |  | antisense | GCUUUCCUUGGCACUAUAC dTdT |
|  | siRNA 2 | sense | UAGUGCCAAGGAAAGCAGC UU |
|  |  | antisense | GCUGCUUUCCUUGGCACUA UU |
|  | siRNA 3 | sense | GUGCCAAGGAAAGCAGCUG UU |
|  |  | antisense | CAGCUGCUUUCCUUGGCAC UU |

**Table S7 Sequences of sh-circRNA 100146**

| **Gene** | **Sequence** |
| --- | --- |
| circRNA 100146-F | GATCCGCCAGTATAGTGCCAAGGAATTCAAGAGATTCCTTGGCACTATACTGGTTTTTTGGAAG |
| circRNA 100146-R | AATTCTTCCAAAAAACCAGTATAGTGCCAAGGAATCTCTTGAATTCCTTGGCACTATACTGGCG |

**Table S8 Sequences of probes used in the FISH experiment**

| **Gene name** | **Label** | **Probe sequence** |
| --- | --- | --- |
| circRNA 100146 | 6-FAM | GGGCCAGCTGCTTTCCTTGGCACTATACTG |
| miR-361-3p | Cy3 | AAATCAGAATCACACCTGGGGGA |
| miR-615-5p | Cy5 | GATCCGAGCACCGGGGACCCCC |

**Table S9 Sequences of probes used in the RNA pull down experiment**

| **Gene name** | **Label** | **Probe sequence** |
| --- | --- | --- |
| circRNA 100146 | Biotin labeling | CCATGAGAGTGGAGAGCTCAACCAGTATAGTGCCAAGGAAAGCAGCTGGCCCTTCTCAAGACCAATTCGGCTGTCCGGACCTGCGGTTTTGACTTTGGGGGCAACATCATCATGTTCTCCACGGACAAGCAGATGGGCTACCAGTGCTTTGTGAGCTTTTTTGACCTGCGGGATCCGAGCCAGATTGACAACAATGAGCCCTACATGAAGATCCCTTGCAATGACTCTAAAATCACCAGTGCTGTTTGGGGACCCCTGGGGGAGTGCATCATCGCTGGCCATGAGAGTGGAGAGCTCAACCAGTATAGTGCCAAG |

**Table S10 Sequences of probes used in the RAP experiment**

| **Gene** | **Probe Sequence** |
| --- | --- |
| circRNA 100146 | AATTGGTCTTGAGAAGGGCCAGCTGCTTTCCTTGGCACTATACTGGTTGAGCTCTCCACT |
| negative control | ATGGCGATGGCTGGTTTCCATCAGTTGCTGTTGACT |

**Table S11 The microRNA data obtained from RAP-sequencing**

| **miRNA ID** | **Total Count** | **Normalized Value** |
| --- | --- | --- |
| hsa-let-7a-5p | 18288 | 1925.6246 |
| hsa-let-7a-3p | 12 | 1.2635 |
| hsa-let-7b-5p | 908 | 95.6073 |
| hsa-let-7b-3p | 13 | 1.3688 |
| hsa-let-7c-5p | 75 | 7.8971 |
| hsa-let-7d-5p | 398 | 41.9072 |
| hsa-let-7d-3p | 31 | 3.2641 |
| hsa-let-7e-5p | 272 | 28.6401 |
| hsa-let-7f-5p | 18027 | 1898.1428 |
| hsa-miR-16-5p | 38 | 4.0012 |
| hsa-miR-16-1-3p | 14 | 1.4741 |
| hsa-miR-17-5p | 161 | 16.9524 |
| hsa-miR-19a-3p | 11 | 1.1582 |
| hsa-miR-20a-5p | 217 | 22.8489 |
| hsa-miR-21-5p | 18326 | 1929.6258 |
| hsa-miR-21-3p | 22 | 2.3165 |
| hsa-miR-22-3p | 289 | 30.4301 |
| hsa-miR-23a-3p | 247 | 26.0077 |
| hsa-miR-24-3p | 1380 | 145.3063 |
| hsa-miR-24-2-5p | 24 | 2.5271 |
| hsa-miR-25-3p | 866 | 91.185 |
| hsa-miR-26a-5p | 1463 | 154.0458 |
| hsa-miR-26b-5p | 73 | 7.6865 |
| hsa-miR-27a-5p | 13 | 1.3688 |
| hsa-miR-27a-3p | 741 | 78.0232 |
| hsa-miR-28-3p | 100 | 10.5294 |
| hsa-miR-29a-3p | 166 | 17.4789 |
| hsa-miR-30a-5p | 1531 | 161.2058 |
| hsa-miR-30a-3p | 277 | 29.1666 |
| hsa-miR-31-5p | 18 | 1.8953 |
| hsa-miR-92a-3p | 4070 | 428.5484 |
| hsa-miR-93-5p | 220 | 23.1648 |
| hsa-miR-96-5p | 26 | 2.7377 |
| hsa-miR-98-5p | 273 | 28.7454 |
| hsa-miR-100-5p | 5405 | 569.1164 |
| hsa-miR-101-3p | 138 | 14.5306 |
| hsa-miR-29b-3p | 16 | 1.6847 |
| hsa-miR-103a-3p | 742 | 78.1285 |
| hsa-miR-106a-5p | 33 | 3.4747 |
| hsa-miR-192-5p | 96 | 10.1083 |
| hsa-miR-196a-5p | 161 | 16.9524 |
| hsa-miR-197-3p | 28 | 2.9482 |
| hsa-miR-199a-5p | 11 | 1.1582 |
| hsa-miR-199a-3p | 13 | 1.3688 |
| hsa-miR-129-5p | 45 | 4.7382 |
| hsa-miR-148a-3p | 3390 | 356.9481 |
| hsa-miR-30c-5p | 263 | 27.6924 |
| hsa-miR-30c-2-3p | 25 | 2.6324 |
| hsa-miR-30d-5p | 4106 | 432.339 |
| hsa-miR-7-5p | 729 | 76.7596 |
| hsa-miR-10a-5p | 48 | 5.0541 |
| hsa-miR-10b-5p | 19 | 2.0006 |
| hsa-miR-181a-5p | 26 | 2.7377 |
| hsa-miR-181a-2-3p | 29 | 3.0535 |
| hsa-miR-181b-5p | 73 | 7.6865 |
| hsa-miR-182-5p | 3022 | 318.1998 |
| hsa-miR-183-5p | 2275 | 239.5448 |
| hsa-miR-203a-3p | 199 | 20.9536 |
| hsa-miR-205-5p | 1733 | 182.4753 |
| hsa-miR-210-3p | 31 | 3.2641 |
| hsa-miR-215-5p | 28 | 2.9482 |
| hsa-miR-218-5p | 11 | 1.1582 |
| hsa-miR-221-5p | 69 | 7.2653 |
| hsa-miR-221-3p | 299 | 31.483 |
| hsa-miR-222-3p | 2469 | 259.972 |
| hsa-miR-200b-3p | 205 | 21.5854 |
| hsa-let-7g-5p | 2505 | 263.7626 |
| hsa-let-7i-5p | 6006 | 632.3984 |
| hsa-miR-1-3p | 35 | 3.6853 |
| hsa-miR-15b-5p | 14 | 1.4741 |
| hsa-miR-23b-3p | 45 | 4.7382 |
| hsa-miR-27b-3p | 304 | 32.0095 |
| hsa-miR-122-5p | 34 | 3.58 |
| hsa-miR-125b-5p | 92 | 9.6871 |
| hsa-miR-125b-1-3p | 18 | 1.8953 |
| hsa-miR-128-3p | 315 | 33.1677 |
| hsa-miR-140-3p | 19 | 2.0006 |
| hsa-miR-141-3p | 22 | 2.3165 |
| hsa-miR-143-3p | 75 | 7.8971 |
| hsa-miR-152-3p | 81 | 8.5288 |
| hsa-miR-191-5p | 883 | 92.975 |
| hsa-miR-9-5p | 32 | 3.3694 |
| hsa-miR-125a-5p | 247 | 26.0077 |
| hsa-miR-126-3p | 53 | 5.5806 |
| hsa-miR-149-5p | 22 | 2.3165 |
| hsa-miR-185-5p | 241 | 25.376 |
| hsa-miR-186-5p | 10 | 1.0529 |
| hsa-miR-320a | 1759 | 185.2129 |
| hsa-miR-200c-3p | 5441 | 572.907 |
| hsa-miR-155-5p | 37 | 3.8959 |
| hsa-miR-106b-3p | 140 | 14.7412 |
| hsa-miR-200a-5p | 12 | 1.2635 |
| hsa-miR-34c-5p | 33 | 3.4747 |
| hsa-miR-99b-5p | 1516 | 159.6264 |
| hsa-miR-99b-3p | 24 | 2.5271 |
| hsa-miR-130b-5p | 15 | 1.5794 |
| hsa-miR-30e-5p | 71 | 7.4759 |
| hsa-miR-30e-3p | 37 | 3.8959 |
| hsa-miR-361-5p | 14 | 1.4741 |
| hsa-miR-361-3p | 59 | 6.2124 |
| hsa-miR-378a-3p | 1157 | 121.8257 |
| hsa-miR-340-5p | 22 | 2.3165 |
| hsa-miR-330-3p | 24 | 2.5271 |
| hsa-miR-342-3p | 18 | 1.8953 |
| hsa-miR-151a-5p | 44 | 4.633 |
| hsa-miR-151a-3p | 1706 | 179.6323 |
| hsa-miR-148b-3p | 169 | 17.7948 |
| hsa-miR-335-3p | 74 | 7.7918 |
| hsa-miR-196b-5p | 20 | 2.1059 |
| hsa-miR-423-5p | 46 | 4.8435 |
| hsa-miR-423-3p | 1125 | 118.4562 |
| hsa-miR-425-5p | 32 | 3.3694 |
| hsa-miR-451a | 219 | 23.0595 |
| hsa-miR-484 | 26 | 2.7377 |
| hsa-miR-486-5p | 81 | 8.5288 |
| hsa-miR-486-3p | 46 | 4.8435 |
| hsa-miR-146b-5p | 125 | 13.1618 |
| hsa-miR-181d-5p | 17 | 1.79 |
| hsa-miR-500a-3p | 19 | 2.0006 |
| hsa-miR-503-5p | 26 | 2.7377 |
| hsa-miR-532-5p | 44 | 4.633 |
| hsa-miR-455-5p | 32 | 3.3694 |
| hsa-miR-92b-3p | 126 | 13.2671 |
| hsa-miR-582-3p | 11 | 1.1582 |
| hsa-miR-584-5p | 241 | 25.376 |
| hsa-miR-615-5p | 73 | 7.6865 |
| hsa-miR-619-5p | 12 | 1.2635 |
| hsa-miR-629-5p | 24 | 2.5271 |
| hsa-miR-671-3p | 27 | 2.8429 |
| hsa-miR-320c | 48 | 5.0541 |
| hsa-miR-769-5p | 12 | 1.2635 |
| hsa-miR-675-5p | 29 | 3.0535 |
| hsa-miR-744-5p | 227 | 23.9018 |
| hsa-miR-877-5p | 16 | 1.6847 |
| hsa-miR-374b-5p | 13 | 1.3688 |
| hsa-miR-760 | 22 | 2.3165 |
| hsa-miR-941 | 20 | 2.1059 |
| hsa-miR-1180-3p | 10 | 1.0529 |
| hsa-miR-1290 | 413 | 43.4866 |
| hsa-miR-1291 | 10 | 1.0529 |
| hsa-miR-1304-3p | 24 | 2.5271 |
| hsa-miR-1246 | 1295 | 136.3563 |
| hsa-miR-1257 | 11 | 1.1582 |
| hsa-miR-1261 | 56 | 5.8965 |
| hsa-miR-1268a | 48 | 5.0541 |
| hsa-miR-1275 | 23 | 2.4218 |
| hsa-miR-1307-5p | 13 | 1.3688 |
| hsa-miR-1307-3p | 600 | 63.1767 |
| hsa-miR-103b | 11 | 1.1582 |
| hsa-miR-3184-5p | 367 | 38.6431 |
| hsa-miR-3184-3p | 269 | 28.3242 |
| hsa-miR-320e | 11 | 1.1582 |
| hsa-miR-3195 | 36 | 3.7906 |
| hsa-miR-3607-3p | 13 | 1.3688 |
| hsa-miR-3615 | 67 | 7.0547 |
| hsa-miR-3653-3p | 18 | 1.8953 |
| hsa-miR-3687 | 35 | 3.6853 |
| hsa-miR-3934-5p | 19 | 2.0006 |
| hsa-miR-4448 | 14 | 1.4741 |
| hsa-miR-3135b | 12 | 1.2635 |
| hsa-miR-4488 | 34 | 3.58 |
| hsa-miR-4492 | 18 | 1.8953 |
| hsa-miR-4497 | 85 | 8.95 |
| hsa-miR-4500 | 66 | 6.9494 |
| hsa-miR-4508 | 20 | 2.1059 |
| hsa-miR-4516 | 10 | 1.0529 |
| hsa-miR-3960 | 50 | 5.2647 |
| hsa-miR-3529-3p | 1711 | 180.1588 |
| hsa-miR-4791 | 31 | 3.2641 |
| hsa-miR-1273g-3p | 15 | 1.5794 |
| hsa-miR-892c-5p | 10 | 1.0529 |
| hsa-miR-6747-3p | 16 | 1.6847 |
| hsa-miR-7641 | 39 | 4.1065 |
| hsa-miR-7704 | 61 | 6.423 |
| hsa-miR-7706 | 43 | 4.5277 |
| hsa-miR-7974 | 20 | 2.1059 |
