# Supplementary material for: Circular RNA 100146 functions as an oncogene through direct binding to miR-361-3p and miR-615-5p in non-small cell lung cancer
Source: Mol Cancer. 2019 Jan 21;18:13. doi: 10.1186/s12943-019-0943-0 (PMC6340182; doi:10.1186/s12943-019-0943-0)
Supplement: Supplementary file 4 — Figure S1. Identification of circRNA 100146 and its expression in lung cancer tissues. Figure S2. Suppression of circRNA 100146 expresssion inhibits cancer cell invasion and migration in vitro. Figure S3. circRNA 100146 binds subtypes of splicing factor SF3 family. Figure S4. circRNA 100146 binding to miR-361-3p and miR-615-5p indirectly affecting multiple downstream mRNAs expression. (DOCX 4346 kb) [file 12943_2019_943_MOESM4_ESM.docx]

**Supplementary Figures and Legends**

**Figure S1**

**
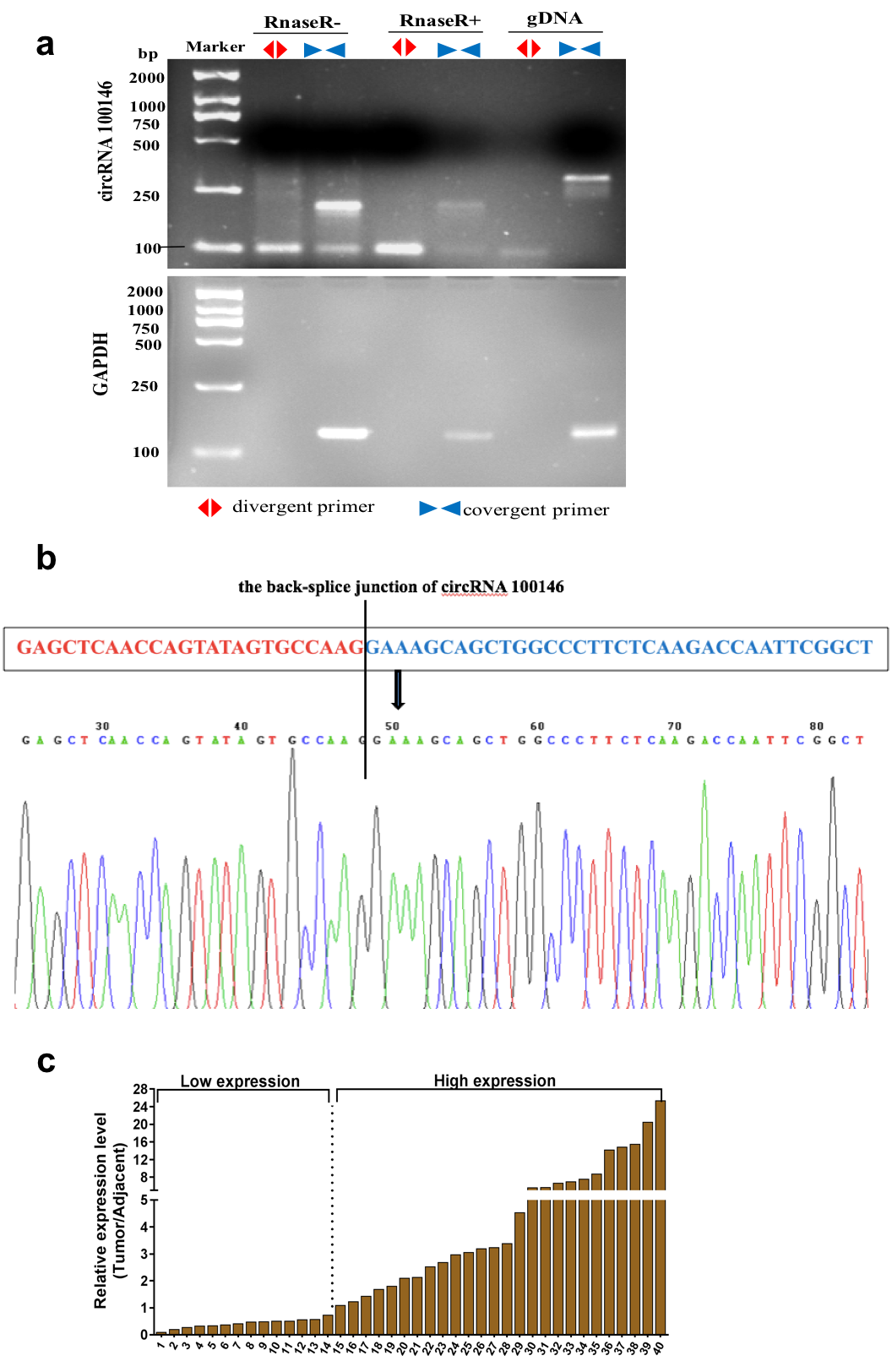
**

**Figure S1.** Identification of circRNA 100146 and its expression in lung cancer tissues. **(a)** circRNA 100146 was detected in cDNA and gDNA by using divergent primer and convergent primer in16HBE cells, respectively. When nuclease is not used, the circRNA products band can be detected in the cDNA using the divergent primer, and the band detected by the convergent primer is nonspecific. When RNA was treated with nuclease, the corresponding band of circRNA can also be detected by the divergent primer, but the convergent primer was not detected. The corresponding band of circRNA was not detected by the divergent primer in the gDNA. *GAPDH* as a control. These indicate that the circRNA is formed by back-splicing and resistant to RNase R. **(b)** Sanger sequencing (lower part of result ) confirms head-to-tail splicing of circRNA 100146. **(c)**Relative expression of the circRNA in NSCLC and matched paracancerous tissues. Y axis represents the ratio of circRNA 100146 expression in cancer (2^-ΔCt) and paracancerous tissue (2^-ΔCt)，and the ratio >1 indicates a high-expression of circRNA 100146.

**Figure S2**


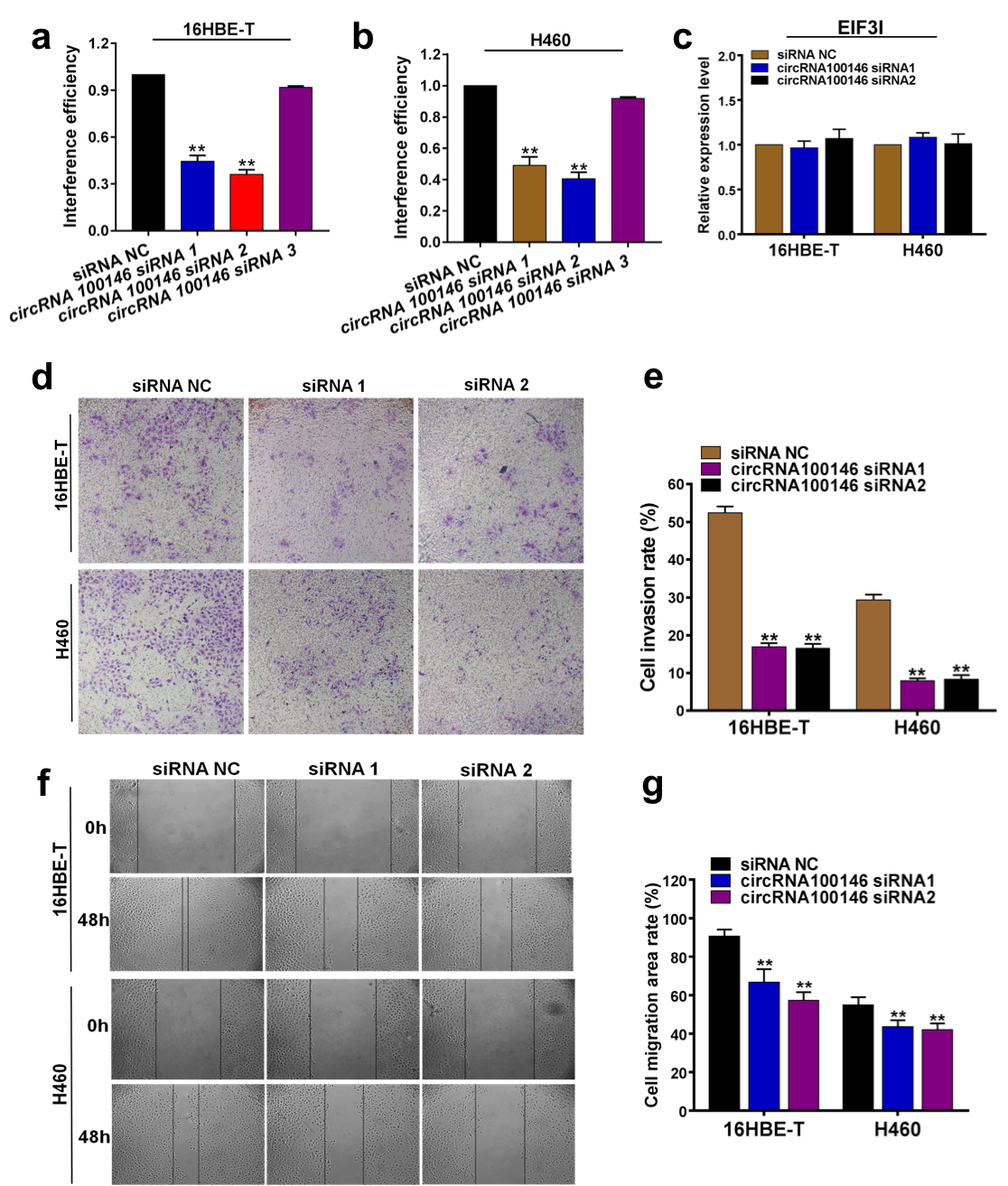


**
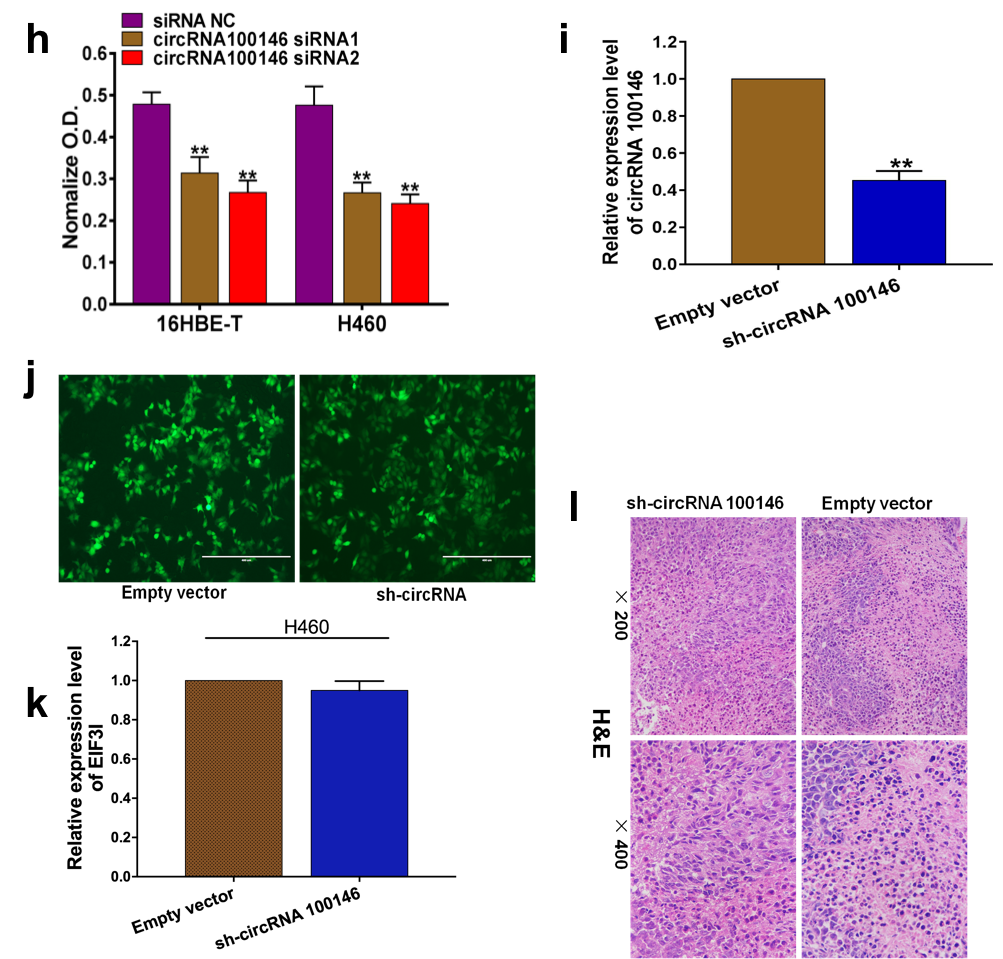
**

**Figure S2. Suppression of circRNA 100146 expresssion inhibits cancer cell invasion and migration *in vitro*. (a)** qRT-PCR of the interference efficiency of three specific siRNAs for circRNA 100146 in 16HBE-T cells. Compared with siRNA NC, relative expression of circRNA100146 was reduced to 0.45±0.06 and 0.36±0.05 after transfection of cells with siRNA1 and siRNA2, respectively. **(b)** qRT-PCR analysis of the interference efficiency of three specific siRNAs of circRNA 100146 in H460 cells. Compared with siRNA NC, relative expression of circRNA100146 was reduced to 0.49 ± 0.09 and 0.40 ± 0.07, respectively with siRNA1 and siRNA2. **(c)** *EIF3I* is the parental gene of circRNA100146. After siRNA-mediated knockdown of circRNA 100146 in 16HBE-T and H460 cells, *EIF3I* mRNA expression was detected unchanged via qRT-PCR. **(d)** The transwell assay was used to detect 16HBE-T and H460 cell migration (magnification × 200). **(e)** Five vision fields were randomly selected in each group and cells counted to determine the invasion rate. **(f)** The wound healing assay was performed in 16HBE-T and H460 cells. **(g)** Ratios of migration areas of 16HBE-T and H460 cells were analyzed with Image J software. **(h)** Cell adhesion was detected with the CCK-8 assay after 48 h of interference. The absorbance of each well was measured with a microplate reader at 450 nm. **(i)** qRT-PCR analysis of the relative expression of circRNA 100146 in H460-empty vector and H460 sh-circRNA groups, ** *p*<0.01. **(j)** Images of H460 cell groups stably transfected with empty vector and sh-circRNA were obtained under a fluorescence microscope. **(k)**The relative expression of *EIF3I* was detected in H460-empty vector and H460 sh-circRNA groups. **(l)** HE staining of subcutaneous tumors (Magnification, × 200 and × 400). **(a, b, c, e, g, h, i, k)** Data are represented as means ± s.d., n = 3, unpaired t-tests; compared with siRNA NC or empty-vector group, ** *p*<0.01.

**Figure S3**

**
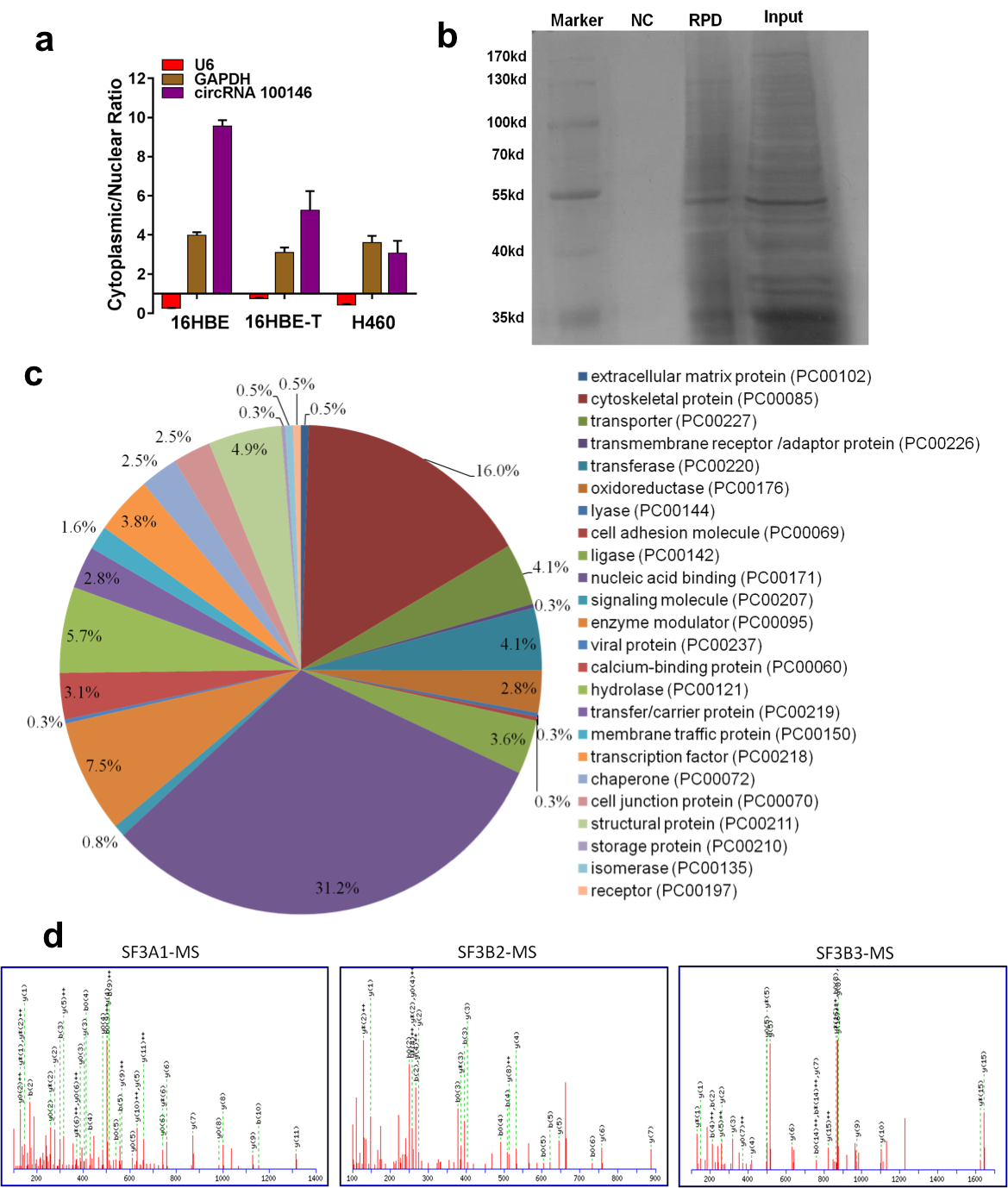
**

**Figure S3. circRNA 100146 binds subtypes of splicing factor SF3 family. (a)** Relative circRNA 100146 expression at the subcellular level was detected via qRT-PCR. U6 and GAPDH were used as the internal control genes and the cytoplasm/nucleus ratio measured as 2 ^ ^(cytoplasm CT value - nucleus CT value)^. U6 was mainly expressed in the nucleus which the cytoplasm/nucleus ratio was close to 0. The proportion of GAPDH expressed in the cytoplasm was 75%-80% and that of cytoplasmic circRNA of 16HBE, 16HBE-T and H460 cells was 95.60%, 87.61% and 76.75%, respectively. **(b)** SDS-PAGE silver staining of circRNA 100146 pull-down samples in H460 cells. NC represents negative control. The RPD group contained a circRNA 100146 specific pull-down probe. Input represents the positive control. **(c)** Categories of proteins obtained via the pull-down experiment and identified using protein mass spectrometry. **(d)** Representative mass spectral maps of splicing factors SF3A1, SF3B2, and SF3B3.

**Figure S4**

**
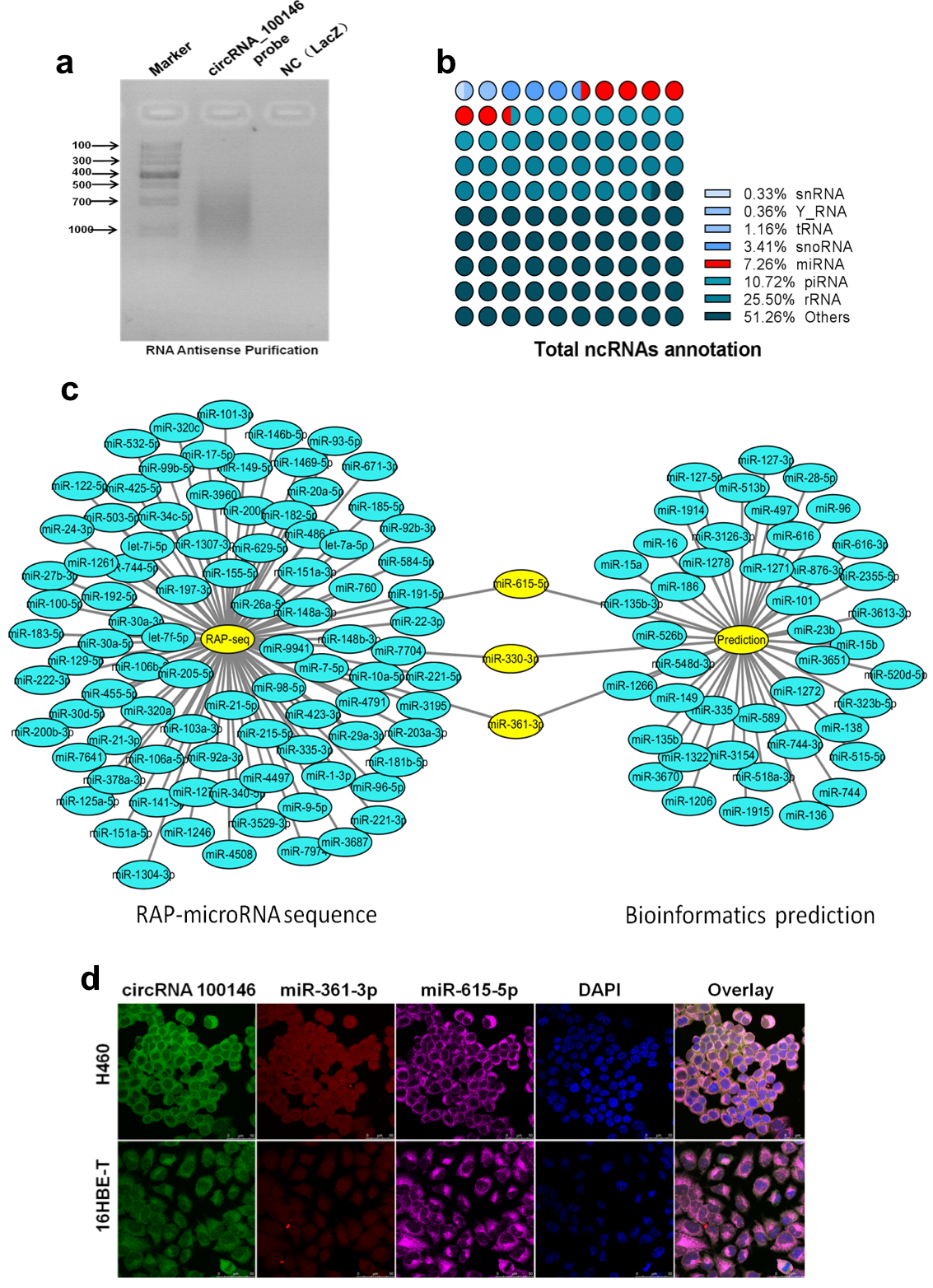
**


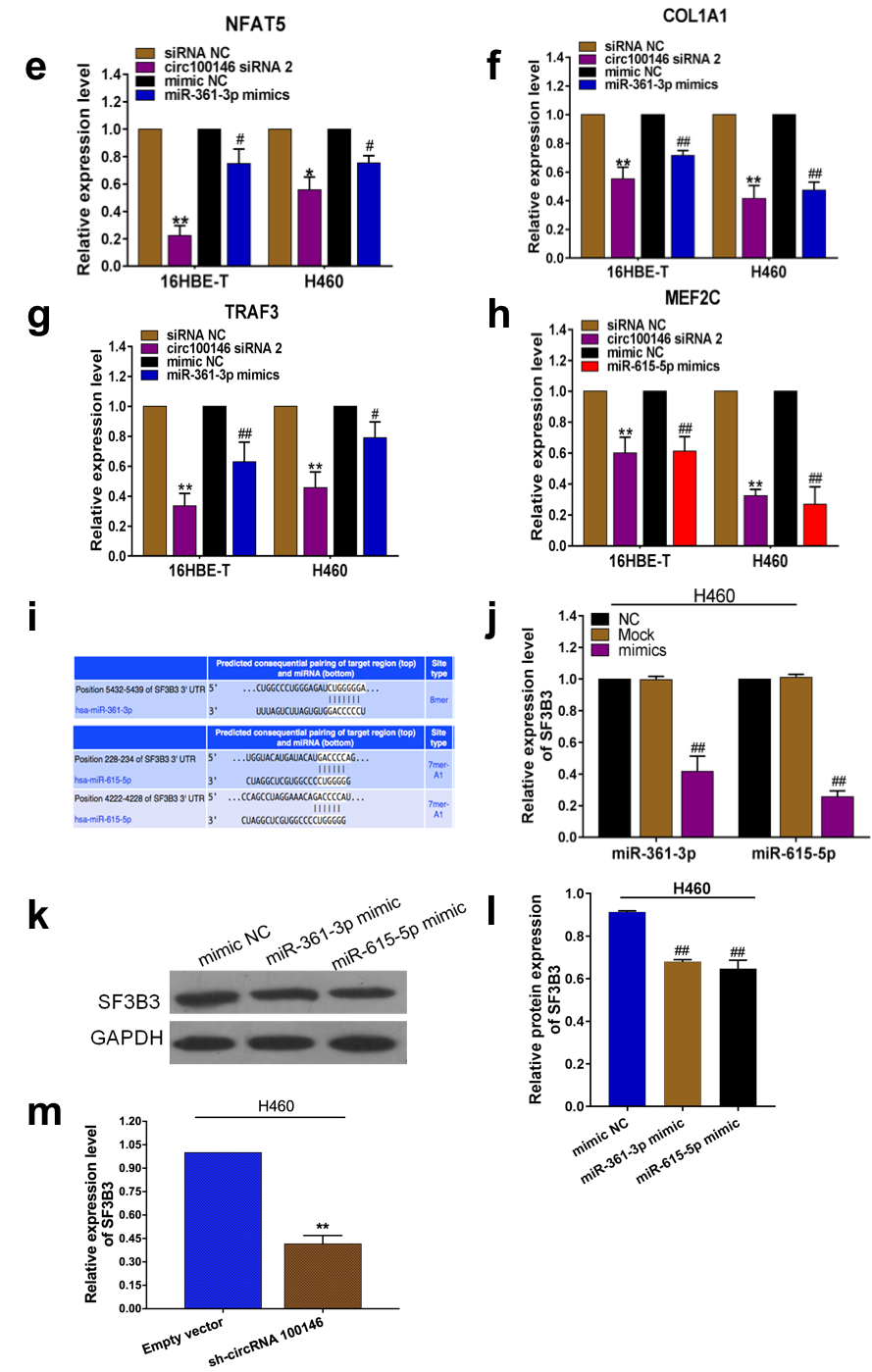


**Figure S4. circRNA 100146 binding to miR-361-3p and miR-615-5p indirectly affecting multiple downstream mRNAs expression.**

**(a)** RNA antisense purification electrophoresis map. **(b)**Percentages of various non-coding RNAs estimated via small RNA sequencing. **(c)** Screening target miRNAs of circRNA 100146 from those obtained via sequencing and those predicted using bioinformatics. **(d)** Subcellular localization of circRNA 100146, miR-361-3p and miR-615-5p in 16HBE-T and H460 cells detected with FISH and observed under a confocal fluorescence microscope. Green fluorescence represents 6-FAM-labeled circRNA probe, red represents the Cy3-labeled miR-361-3p probe, purple represents the Cy5-labeled miR-615-5p probe, and blue represents DAPI-stained nuclei. **(e-g)** qRT-PCR analysis of the relative expression of *NFAT5* **(e)**, *COL1A1* **(f)** and *TRAF3* **(g)** upon downregulation of circRNA 100146 or overexpression of miR-361-3p. **(h)** Relative expression of *MEF2C* upon downregulation of circRNA 100146 or over-expression of miR-615-5p. **(i)** Binding sites of miR-361-3p and miR-615-5p in the 3'-UTR regions of SF3B3 predicted by TargetScan. **(j)** Following overexpression of miR-361-3p and miR-615-5p in H460 cells, *SF3B3* expression was detected via qRT-PCR. NC represents the mimic negative control group and Mock represents the transfection reagent control group. **(k)** Following overexpression of miR-361-3p and miR-615-5p in H460 cells, SF3B3 protein expression was detected via western blot. **(l)** Gray value analysis of protein bands in **(k)**. **(m)** The relative expression of *SF3B3* was detected in H460-empty vector and H460 sh-circRNA groups. **(e, f, g, h, j, l, m)** Data are presented as means±s.d., n = 3, unpaired *t*-tests; compared with siRNA NC or empty-vector group , **p*<0.05, ***p*<0.01; compared with the mimic NC, # *p*<0.05, ##*p*<0.01.
